# Benthic macrofaunal structure and secondary production in tropical estuaries on the Eastern Marine Ecoregion of Brazil

**DOI:** 10.7717/peerj.4441

**Published:** 2018-02-28

**Authors:** Lorena B. Bissoli, Angelo F. Bernardino

**Affiliations:** Grupo de Ecologia Bêntica, Department of Oceanography, Universidade Federal do Espírito Santo, Vitoria, ES, Brazil

**Keywords:** Estuaries, Benthic ecology, Secondary production, Mangroves, Macrofauna, Eastern Brazil, Tidal flats

## Abstract

Tropical estuaries are highly productive and support diverse benthic assemblages within mangroves and tidal flats habitats. Determining differences and similarities of benthic assemblages within estuarine habitats and between regional ecosystems may provide scientific support for management of those ecosystems. Here we studied three tropical estuaries in the Eastern Marine Ecoregion of Brazil to assess the spatial variability of benthic assemblages from vegetated (mangroves) and unvegetated (tidal flats) habitats. A nested sampling design was used to determine spatial scales of variability in benthic macrofaunal density, biomass and secondary production. Habitat differences in benthic assemblage composition were evident, with mangrove forests being dominated by annelids (Oligochaeta and Capitellidae) whereas peracarid crustaceans were also abundant on tidal flats. Macrofaunal biomass, density and secondary production also differed between habitats and among estuaries. Those differences were related both to the composition of benthic assemblages and to random spatial variability, underscoring the importance of hierarchical sampling in estuarine ecological studies. Given variable levels of human impacts and predicted climate change effects on tropical estuarine assemblages in Eastern Brazil, our data support the use of benthic secondary production to address long-term changes and improved management of estuaries in Eastern Brazil.

## Introduction

Estuaries are productive ecosystems that commonly support large densities and biomass of benthic organisms (Kennish, 2002). The benthic macrofauna has an important role on estuarine productivity through sediment bioturbation, trophic linkages and facilitating biogeochemical processes (Ysebaert et al., 1998; Herman et al., 1999; Nilsen, Pedersen & Nilssen, 2006; Kristensen & Kostka, 2005; Kristensen, 2008; Kristensen et al., 2014). Given the strong linkage between benthic dynamics and estuarine ecosystem functioning, spatial and temporal changes in sediment composition and organic matter between estuarine habitats are of interest to understand ecosystem productivity (Edgar & Barrett, 2002; Kristensen et al., 2014; Morais, Camargo & Lana, 2016).

Spatial patterns of macrofaunal assemblages reflect factors that act at a number of spatial scales (Edgar & Barrett, 2002; Barros et al., 2008; Blanchet et al., 2014; Giménez et al., 2014). Sediment grain size, organic matter quality and quantity, plant cover, and disturbance (e.g., hydrology) are frequently associated with macrofaunal spatial heterogeneity in estuaries. Spatial changes in macrofaunal assemblages that occur between vegetated and unvegetated estuarine habitats have also been previously quantified in some areas (Lana & Guiss, 1991; Edgar et al., 1994; Netto & Lana, 1997; Checon et al., 2017; Bernardino et al., 2018). Although an increased abundance, biomass and production of macrofauna has been reported for estuarine vegetated habitats (Lana & Guiss, 1991; Heck et al., 1995; Sheridan, 1997; Dolbeth et al., 2007; Ponti, Colangelo & Ceccherelli, 2007; Kon, Kurokura & Tongnunui, 2010), patterns of benthic diversity and assemblage composition have been less clearly associated with differences in habitat.

Benthic secondary production is an important ecological parameter to understand ecosystem dynamics as it allows energy flow estimates within ecosystems and represents the formation of community biomass by growth through time (Dolbeth et al., 2005; Dolbeth et al., 2012; Benke, 2010). Benthic secondary production is an indicator of both population dynamics (biomass, life span and body-size) and also biotic interactions and environmental variability within ecosystems (Waters & Crawford, 1973; Dolbeth et al., 2012). These indicators vary with estuarine environmental changes and therefore influence secondary production. For example, temperature can influence growth rates and reproduction, leading to an increase in production in warmer waters (Tumbiolo & Downing, 1994). So, changes in water temperature, nutrient and oxygen availability, and also habitat heterogeneity including variations in sediment grain size and vegetation are likely to have an effect on secondary production (Edgar et al., 1994; Heck et al., 1995; Edgar & Barrett, 2002; Dolbeth et al., 2003; Rodrigues et al., 2006). Benthic secondary production can therefore be used to represent functional responses of assemblages subjected to long-term environmental and local anthropogenic impacts (Benke, 2010; Dolbeth et al., 2011; Dolbeth et al., 2012).

The spatial patterns of secondary production in mangroves and unvegetated estuarine tidal flats are largely unknown, especially for tropical estuaries (Alongi, 2002; Lee, 2008). In South America, although the Brazilian coast has hundreds of estuarine systems, benthic production has only been evaluated on epibenthic assemblages (i.e., crabs and gastropods) in the Amazon Ecoregion, or focused on specific populations in some localities (Pagliosa & Lana, 2000; Koch & Wolff, 2002; Costa & Soares-Gomes, 2015; Bernardino et al., 2016). Given the increasing human and climatic impacts on estuarine ecosystems, understanding spatial patterns of estuarine benthic secondary production may be invaluable to monitoring and conservation of these ecosystems (Alongi, 2002; Kennish, 2002). This study investigated benthic secondary production, biomass and density at variable spatial scales in vegetated and unvegetated habitats from three tropical estuaries in the Eastern Brazil Marine Ecoregion. We tested the hypothesis that spatial variations in benthic communities occurs between vegetated and unvegetated habitats (scales of habitat) and among estuaries (scales of estuary). We expected to find higher production of benthic fauna within mangrove forests in response to higher organic availability and higher faunal biomass when compared to unvegetated tidal flat habitats.

## Material & Methods

### Study area

**Figure 1:**
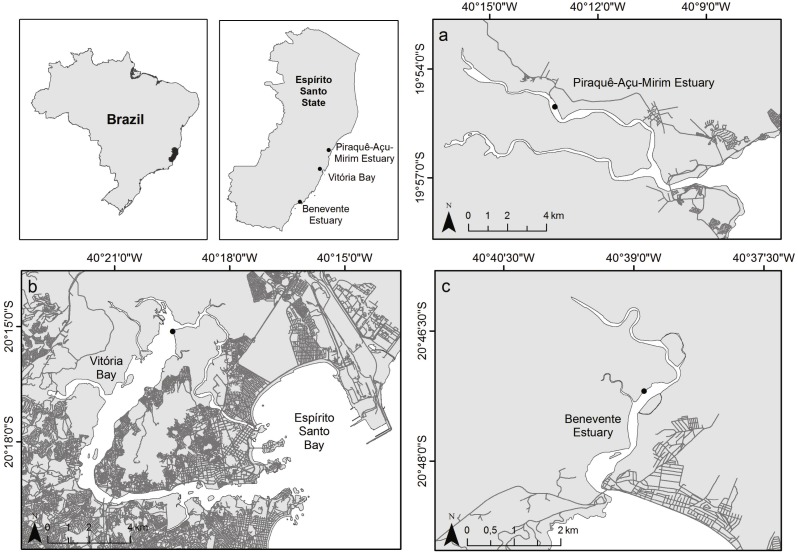
Map of study sites. Study area indicating the three sampled estuaries. (A) Piraquê-Açu-Mirim estuary (PAE), (B) Vitória Bay (VIB), (C) Benevente estuary (BEN).

The study was carried out in three tropical estuaries with a microtidal, semidiurnal tidal pattern within the Eastern Brazil Marine Ecoregion (Spalding et al., 2007; Fig. 1). The northernmost estuary, Piraquê-Açu-Mirim estuary (PAE; 19°57′S 40°09′W) is within a municipal conservation unit and covered by extensive and well-developed mangrove forests with an area of over 19 km^2^ (Bernardino et al., 2018; Servino, Gomes & Bernardino, 2018). The Vitória Bay estuarine system (VIB; 20°16′S 40°20′W) is the largest estuary in the region with approximately 18 km^2^ of mangrove forests and surrounded by a densely populated metropolitan area with high levels of sewage input and industrial activities (Jesus et al., 2004). The southernmost estuary, Benevente estuary (BEN, 20°48′S 40°39′W), has well preserved mangrove forests that cover an area of approximately 4.6 km^2^ with minor urban settlement (Pereira et al., 2009; Petri et al., 2011). Mangrove forests of the three estuaries are composed by *Rhizophora mangle*, *Laguncularia racemosa* and *Avicennia schaueriana* species.

### Sampling and sample processing

Benthic macrofaunal assemblages were sampled on a nested spatial design on vegetated (V—mangrove forests) and unvegetated (NV—tidal flats) habitats on the mesohaline sectors (salinity range between 18 and 5; Venice system, 1958) of the three estuaries (VIB, PAE and BEN, ICMBIO permit N 24700-1). Salinity sectors in the sampled estuaries were measured with either a multiparameter or with semi-continuous (5–20 days) deployment of data-loggers. Sampling occurred in one sampling event in each estuary between August and September 2014, during low tides and on the dry season. Each estuary was divided in two sites distanced in the scale of hundreds of meters containing adjacent vegetated and unvegetated habitats (Fig. A1). Three sampling plots were randomly established in each habitat and site, parallel to the waterline and separated by tens of meters. Three replicate faunal samples were sampled within each plot, distanced by approximately 1 m from each other using a PVC corer with 15 cm diameter (0.0177 m^2^ area) and to a sediment depth of 10 cm. Additionally, one composite sediment sample was collected at each plot for sediment analysis (grain size, total organic matter and chlorophyll-a), by mixing three samples of 7 cm diameter and 5 cm depth. Superficial water temperature and salinity were measured in each sampling area.

Faunal samples were preserved in 4% formalin and posteriorly washed through a 1 mm sieve and the retained material was stored in 70% ethanol. In the laboratory, samples were sieved through a stacked series of sieves (1, 1.4, 2, 2.8 and 4 mm), using the methods described by Edgar (1990a). Macrofauna was sorted in each sieve size and identified at family level, considering that this level of identification is satisfactory to identify differences in macrofaunal assemblages for the aims of this study (Warwick, 1988; Chapman, 1998; Olsgard, Somerfield & Carr, 1998; De Biasi, Bianchi & Morri, 2003). During sorting of samples, the plant material was separated for plant biomass (plant detritus and living roots) determination (dry weight) after drying at 60 °C during 48 h.

Sediment subsamples were treated with hydrogen peroxide (H_2_O_2_), to eliminate organic matter, and mud content was determined by wet sieving samples through a 0.063 mm mesh size. After drying, the sediment >0.063 mm was sieved through a series of sieves and grain size was classified following the Wentworth scale (Suguio, 1973). Sediment total organic matter (TOM) content was estimated by weight loss after combustion at 500 °C during 4 h. Chlorophyll-*a* (Chl-*a*) and phaeopigments were extracted from the sediment with acetone and analyzed using a spectrophotometer before and after acidification with HCl (Lorenzen, 1967; Quintana et al., 2015).

### Faunal biomass and secondary production

Macrofauna was wet weighed within each taxonomic group, generally family, by each sieve size (1, 1.4, 2, 2.8 and 4 mm) after identification. Macrofaunal biomass (mg wet weight m^−2^) was converted into ash-free dry weight (mg AFDW m^−2^) using the conversion factors compiled in Brey (2001) and Brey et al. (2010). Shells of mollusks were excluded from biomass estimation. Conversion factors from Brey (instead of estimate by methodology used by Edgar (1990a)) were chosen to avoid overestimation of AFDW and consequently of production, mainly in the larger sieve size, since some individuals with elongated bodies and low weights were retained in the sieves.

The secondary production of benthic macrofauna was estimated using the general equation (*P*) = 0.0049∗*B*^0.80^∗*T*^0.89^ of Edgar (1990a), which relates daily macrobenthic production P (µg day^−1^) to ash-free dry weight B (µg) and water temperature T (^∘^C). The water temperature measured in all three estuaries during faunal sampling had a small range so the temperature (T) used in the estimation of benthic secondary production was standardized at 23.5°C. The use of a standardized temperature does not show seasonal, daily or spatial variations, thus limiting secondary production estimates (Edgar, 1990a; Tumbiolo & Downing, 1994). Although with a limited temporal applicability, we used a standardized temperature to indicate the relative secondary production between different estuaries (Edgar, 1993; Edgar & Barrett, 2002). Production was calculated for each taxon (Polychaeta, Oligochaeta, Kalliapseudidae, Other Crustacea, Mollusca and Others) within each sieve size and total production per sample was calculated as the sum of these values. The annual production to biomass ratio (P/B) for each habitat in each estuary was calculated from mean production divided by the mean macrofaunal biomass. This parameter can be considered a measurement of biomass turnover rate (Dolbeth et al., 2012).

### Data analysis

The spatial variability in benthic macrofaunal density, biomass and secondary production were evaluated at multiple spatial scales in different habitats using a nested and orthogonal analysis of variance (ANOVA). Habitat was defined as a fixed factor and orthogonal to spatial factors (estuary, site, plot). Spatial factors were treated as random and included three estuaries, sites (*N* = 2) nested in estuary, plots (*N* = 3) nested in site and samples (*N* = 3) collected at plots. Spatial differences on sediment properties and plant biomass were assessed by ANOVA across scales of estuary, and site (nested in estuary), due to the lack of sample replication at plots. This ANOVA also included habitat factors orthogonal to spatial factors since both vegetated and unvegetated habitats were sampled. A Cochran’s test was performed previously to each ANOVA to assess homogeneity of variances and when necessary data was transformed.

Differences among macrofaunal assemblages were assessed by a Permutational Multivariate Analysis of Variance (PERMANOVA) that was performed using Bray–Curtis similarity coefficients on square-root transformed abundance data (9999 permutations, Anderson, Gorley & Clarke, 2008). A non-metric multidimensional scaling (nMDS) performed using Bray–Curtis similarity matrix and square-root transformed data was used to visualize variation in macrofaunal assemblages. A Similarity Percentage analysis (SIMPER) was used to identify the taxa that most contributed to dissimilarities among habitats. The relationships between sediment properties (TOM, Chl*-a*, Mud, plant biomass) and macrofaunal density were investigated using a Canonical Correspondence Analysis (CCA). In this analysis, the density of the top five dominant taxa (comprising over 90% of total density) was used instead of the complete data. In the CCA, the sum of macrofaunal density data from all replicates was used so that the number of samples from density and sediment properties was the same. Part of statistical analyses were performed in the software R (R Core Team, 2015). The GAD package was used to perform ANOVA analysis (Underwood, 1997; Sandrini-Neto & Camargo, 2014) and the Vegan package was used to perform nMDS and CCA analysis (Oksanen et al., 2017). PERMANOVA was carried out using the software PRIMER 6 with the PERMANOVA + add-on package (Clarke & Gorley, 2006; Anderson, Gorley & Clarke, 2008).

## Results

### Sediment properties and plant material

**Figure 2:**
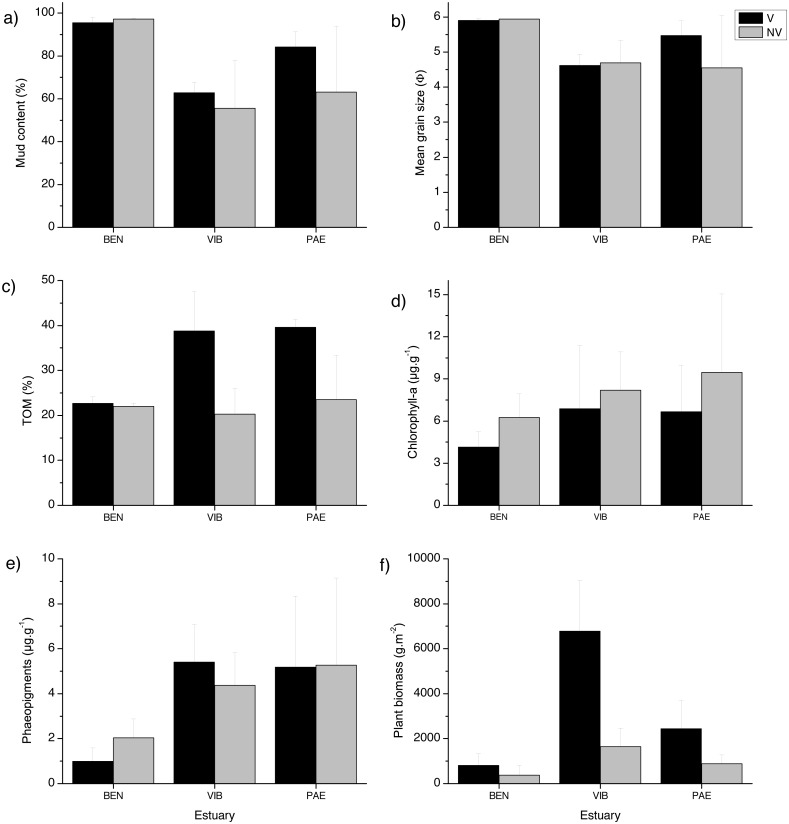
Sediment grain size, Total organic matter, pigments and detritus biomass. Sediment properties and plant material at sampled estuaries. Means (±SD) of (A) mud content (%), (B) mean grain size (Φ), (C) Total Organic Matter (TOM, %^*dw*^), (D) chlorophyll-a (µg g ^−1^), (E) phaeopigments (µg g^−1^) and (F) plant biomass (g^*DW*^ m^−2^). V, vegetated habitat; NV, unvegetated habitat.

The sediment was predominantly composed of mud in all estuaries and habitats (Fig.  2). When comparing the three estuaries, the sediment mud content, mean grain size and total organic matter differed significantly among sites and in the interaction between habitat and site (Table 1). These results represent spatial variation at local scales. Chl*-a* and phaeopigments differed significantly between habitats and estuary, respectively, with higher sediment Chl*-a* at unvegetated habitats and lower phaeopigments in the BEN estuary (Table 1, Fig. 2). Plant biomass differed significantly among estuaries and in the interaction between habitat and site (Table 1). VIB presented over two times higher total plant biomass when compared to similar sectors in the BEN and PAE estuaries (Fig. 2F).

### Macrofaunal density, biomass and secondary production

**Figure 3:**
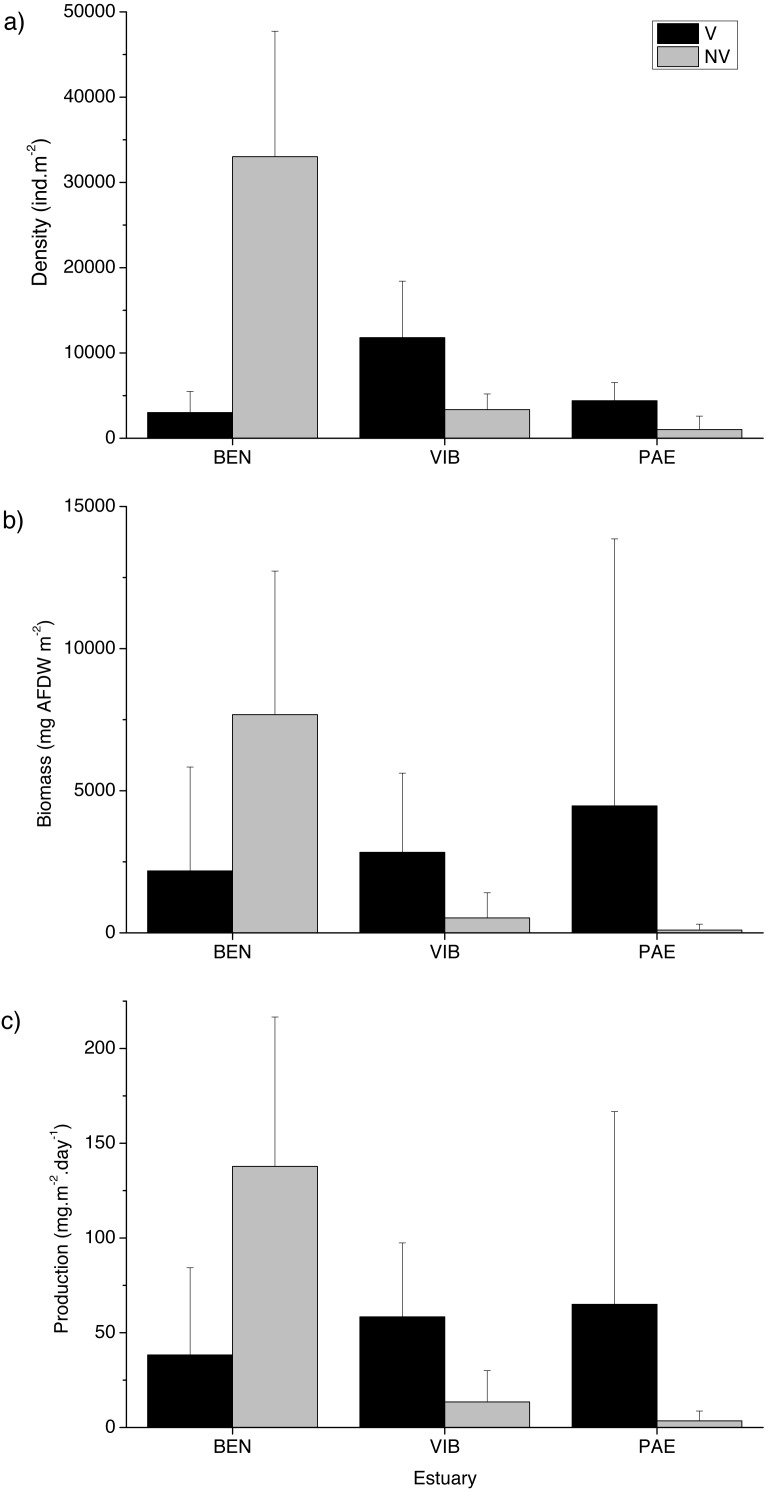
Macrofaunal density, biomass and production. Macrofauna at sampled estuaries. Means (±SD) of macrofaunal (A) density (ind m^−2^), (B) biomass (mg^*AFDW*^ m^−2^) and (C) production (mg m^−2^ day^−1^). V, vegetated habitat; NV, unvegetated habitat.

**Figure 4:**
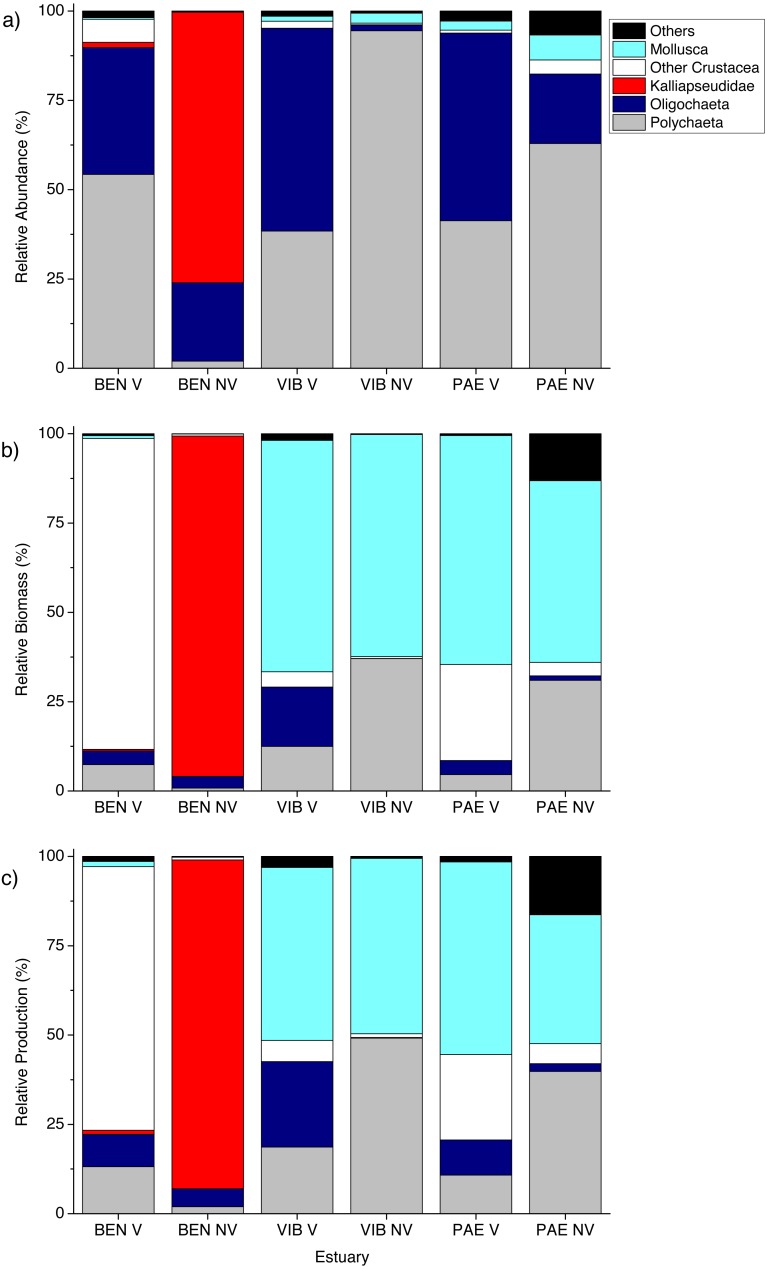
Relative macrofaunal abundance, biomass and production. Relative (A) abundance, (B) biomass and (C) production of macrofauna groups at sampled estuaries. V, vegetated habitat; NV, unvegetated habitat.

A total of 18,036 individuals belonging to 37 taxa were sampled at the three estuaries, of which 7,989 individuals were Kalliapseudidae (Tanaidacea). BEN estuary had a total of 11,481 individuals, distributed in 23 taxa. In PAE estuary 1,728 individuals were collected and distributed in 27 taxa. VIB had a total of 4,827 individuals, distributed in 28 taxa. Within the mesohaline sector of the three estuaries, total macrofaunal density was significantly different at the plot and estuary spatial scales, and in their interactions with habitat (Table  2). The BEN estuary presented higher macrofaunal density in unvegetated habitats, but this pattern was opposite to the VIB and PAE estuaries that had no density differences between habitats (Fig. 3A). Macrofaunal densities varied over 30-fold between unvegetated habitats at BEN and PAE estuaries (33,022 ± 14,709 ind m^−2^ and 1,033 ± 1,558 ind m^−2^, respectively; Fig. 3A). Kalliapseudidae (Tanaidacea) was dominant in unvegetated tidal flats at BEN estuary, whereas Polychaeta and Oligochaeta were more abundant in similar habitats at PAE and VIB estuaries. Vegetated habitats in the three estuaries had higher densities of Oligochaeta and Polychaeta (Fig. 4A).

**Table 1:** ANOVA results for sediment properties and plant material comparing BEN, PAE and VIB estuaries.

Source	*df*	**Mean grain size**			**Mud content**			**TOM**		
		MS	*F*	*p*	MS	*F*	*p*	MS	*F*	*p*
H	1	0.64	0.680	0.50	710.88	1.813	0.31	1,244.64	4.448	0.17
E	2	5.11	3.474	0.17	4,190.30	4.729	0.12	283.85	1.596	0.34
H × E	2	0.95	0.635	0.59	392.01	0.465	0.67	279.85	5.528	0.10
S(E)	3	1.47	6.115	0.003^*^	886.16	8.709	0.0004^*^	177.85	11.505	<0.0001^*^
H × S(E)	3	1.49	6.204	0.003^*^	843.80	8.293	0.0006^*^	50.62	3.275	0.04^*^
Residual	24	0.24			101.75			15.46		

Source	*df*	**Chlorophyll-*a***			**Phaeopigments**			**Plant biomass**		
		MS	*F*	*p*	MS	*F*	*p*	MS	*F*	*p*
H	1	39.04	24.060	0.04^*^	0.06	0.160	0.73	90.50	6.448	0.13
E	2	27.90	1.944	0.29	4.02	479.926	0.0002^*^	79.93	60.220	0.004^*^
H × E	2	1.62	1.447	0.36	0.35	2.915	0.20	14.03	1.727	0.32
S(E)	3	14.35	1.487	0.24	0.01	0.030	0.99	1.33	1.525	0.23
H × S(E)	3	1.12	0.116	0.95	0.12	0.437	0.73	8.13	9.337	0.0003^*^
Residual	24	9.65			0.28			0.87		

**Notes.**

H habitat E estuary S site *df* degrees of freedom MS mean square

* Significant values.

**Table 2:** ANOVA results for macrofaunal density, biomass and secondary production comparing BEN, PAE and VIB estuaries.

		**Density**			**Biomass**			**Secondary production**		
Source	*df*	MS	*F*	*p*	MS	*F*	*p*	MS	*F*	*p*
H	1	311,266.70	0.253	0.66	0.20	0.369	0.61	6.43	0.421	0.58
E	2	689,817.25	19.504	0.02^*^	0.32	4.388	0.13	8.95	5.596	0.10
H × E	2	1,230,614.84	23.040	0.02^*^	0.54	20.178	0.02^*^	15.29	26.162	0.01^*^
S(E)	3	35,368.39	1.372	0.30	0.07	3.427	0.052	1.60	2.982	0.07
P(S(E))	12	25,774.72	4.120	<0.0001^*^	0.02	1.400	0.19	0.54	1.616	0.11
H × S(E)	3	53,413.02	1.431	0.28	0.03	1.061	0.40	0.58	0.990	0.43
H × P(S(E))	12	37,327.63	5.967	<0.0001^*^	0.03	1.682	0.09	0.59	1.779	0.07
Residual	72	6,255.48		0.01		0.33		

**Notes.**

H habitat E estuary S site P plot *df* degrees of freedom MS mean square

* Significant values.

Significant differences in macrofaunal biomass and estimated secondary production were observed only in the interaction between habitat and estuary when comparing the three estuaries (Table 2). Biomass and production followed patterns of macrofaunal density and were not clearly distinct between unvegetated or vegetated habitats among the three estuaries studied (Figs. 3B and 3C). The lowest macrofaunal biomass and production were observed at unvegetated tidal flats in the PAE and VIB estuaries (Figs. 3B and 3C).

**Figure 5:**
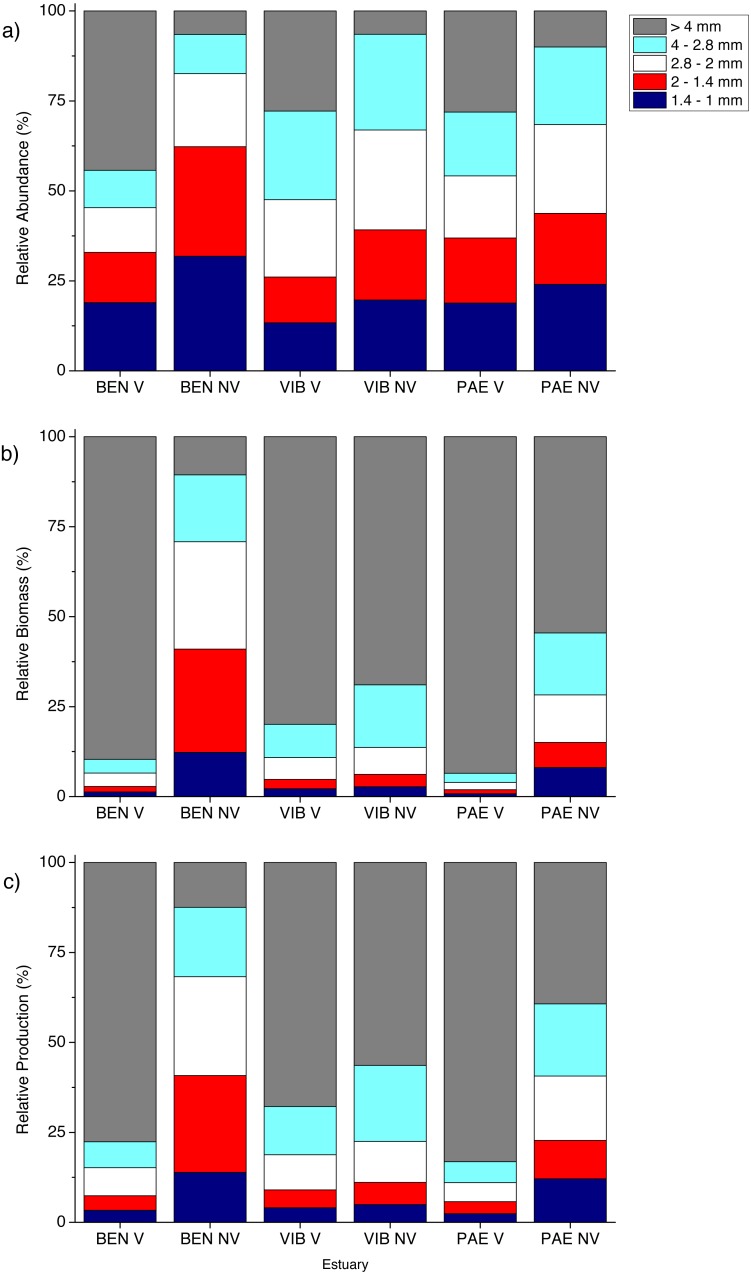
Relative macrofaunal abundance, biomass and production per size classes. Relative (A) abundance, (B) biomass and (C) production of macrofauna size classes at sampled estuaries. V, vegetated habitat; NV, unvegetated habitat.

The contribution from each macrofaunal group to total assemblage biomass and secondary production varied greatly between estuaries and habitats (Fig. 4). Large individuals including bivalve molluscs and brachyuran crabs contributed greatly to benthic biomass and production in vegetated habitats at the three estuaries despite their low density (Figs. 4 and 5). At vegetated habitats in VIB estuary, Mollusca (mainly Mytilidae and Solecurtidae) contributed to most of the biomass (1,832.5 ± 2,780.5 mg AFDW m^−2^) and production (28.3 ± 37.8 mg m^−2^ day^−1^), with Oligochaeta and Polychaeta representing second and third groups respectively. At vegetated habitats of the PAE estuary, Mollusca (mainly Mytilidae; 2,864.6 ± 8,115.6 mg AFDW m^−2^, 35.1 ± 82.9 mg m^−2^ day^−1^) and Crustacea (mainly Panopeidae; 1,199.4 ± 4,331.9 mg AFDW m^−2^, 15.6 ± 49.3 mg m^−2^ day^−1^) were the most representative groups in biomass and production. Crustaceans (mainly Ocypodidae; 1,897.8 ± 3,682.9 mg AFDW m^−2^, 28.3 ± 46.5 mg m^−2^ day^−1^) contributed to over 70% of the macrofaunal biomass and production in vegetated habitats at the BEN estuary with Polychaeta as the second group.

In general, macrofaunal biomass and production of estuarine habitats were mainly derived from large size classes (Figs. 5B and 5C). Vegetated habitats had over 70% of its production from large size classes (>4 mm), whereas unvegetated habitats had more variable contribution (43–87%) of the other size classes from 1 to <4 mm (Figs. 5B and 5C). At unvegetated habitats in the mesohaline sector of VIB (329.4 ± 759.3 mg AFDW m^−2^, 6.6 ± 12.6 mg m^−2^ day^−1^ of Mollusca) and PAE (51.3 ± 193.2 mg AFDW m^−2^, 1.3 ± 4.2 mg m^−2^ day^−1^ of Mollusca) estuaries, Mollusca (mainly Solecurtidae) and Polychaeta (mainly Capitellidae) contributed significantly to total macrofaunal biomass and production (Figs. 4B and 4C). Kalliapseudidae was the dominant taxa at unvegetated habitats in BEN estuary (7,315.7 ± 5,343.6 mg AFDW m^−2^, 126.8 ± 86.8 mg m^−2^ day^−1^) and contributed greatly to biomass and production (over 90%; Figs. 4B and 4C).

The mean estimated community annual production to biomass ratio (P/B) varied among estuaries and habitats. The highest P/B ratio was observed at unvegetated habitats at PAE estuary (12.6 y^−1^), whereas vegetated habitats in this estuary had the lowest P/B ratio (5.3 y^−1^). P/B ratios did not vary significantly between habitats at BEN (6.4 and 6.6 y^−1^ for V and NV respectively) and VIB estuaries (7.5 y^−1^ and 9.3 y^−1^ for V and NV habitats respectively).

### Assemblage composition

Macrofaunal assemblages differed markedly between vegetated and unvegetated habitats and between estuaries (Table 3). The numerically dominant taxa in vegetated habitats in the three estuaries were Oligochaeta and Capitellidae (>90%). In the unvegetated habitats the numerically dominant taxa were more variable among the estuaries. At BEN estuary Kalliapseudidae and Oligochaeta (>98%) were dominant. In unvegetated habitats at VIB Spionidae and Capitellidae (>80%) were more abundant, whereas at PAE estuary Capitellidae and Oligochaeta (75%) dominated in unvegetated habitats. Although differences among the dominant taxa between unvegetated habitats at BEN, VIB and PAE, all three estuaries had most taxa shared between them.

**Table 3:** Mean density (ind m^−2^) and relative abundance (%) of the most representative taxa in vegetated (V) and unvegetated (NV) habitats in areas 1 and 2 (A1 and A2) in the sampled estuaries.

**BEN V**			**BEN NV**		
Taxa	Density (±SD)	Rel. ab. (%)	Taxa	Density (±**SD**)	Rel. ab. (%)
Oligochaeta	1,070 (862)	36	Kalliapseudidae	25,028 (18,207)	76
Capitellidae	728 (501)	24	Oligochaeta	7,235 (13,440)	22
Polychaeta sp1	355 (1,002)	12	Capitellidae	276 (291)	0.8
Ampharetidae	348 (423)	12	Nereididae	182 (181)	0.6
Polychaeta sp2	151 (488)	5	Polychaeta sp1	163 (319)	0.5

**VIB V**			**VIB NV**		
Taxa	Density (±SD)	Rel. abundance	Taxa	Density (±**SD**)	Rel. abundance
Oligochaeta	6,701 (5,356)	57	Spionidae	2,323 (1,628)	69
Capitellidae	2,288 (1,967)	19	Capitellidae	505 (390)	15
Nereididae	1,073 (1,126)	9	Nereididae	248 (250)	7
Spionidae	549 (1,013)	5	Pilargidae	82 (89)	2
Polychaeta sp2	257 (649)	2	Bivalvia não ID	72 (75)	2

**PAE V**			**PAE NV**		
Taxa	Density (±SD)	Rel. abundance	Taxa	Density (±**SD**)	Rel. abundance
Oligochaeta	2,307 (1,510)	53	Capitellidae	512 (949)	50
Capitellidae	1,252 (655)	29	Oligochaeta	201 (320)	19
Nereididae	242 (142)	6	Nemertea	50 (75)	5
Pilargidae	113 (140)	3	Pilargidae	41 (121)	4
Ampharetidae	113 (111)	3	Aoridae	35 (55)	3

**Figure 6:**
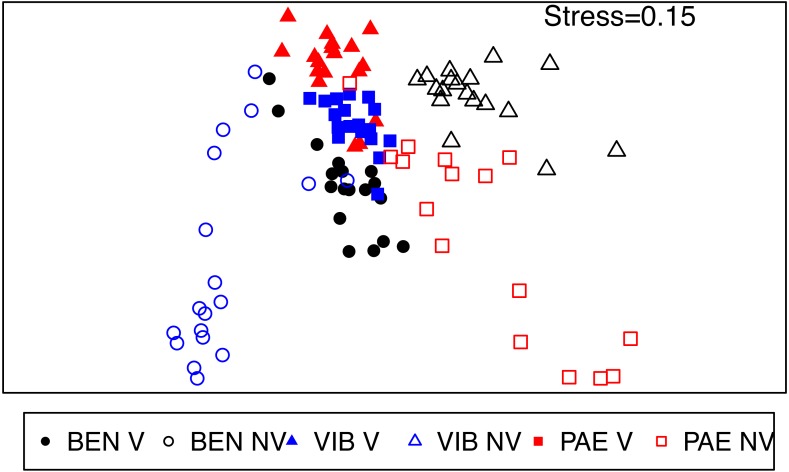
nMDS of macrofaunal assemblages across habitats and estuaries. Non-metric multidimensional scaling (nMDS) ordination plot of community data from vegetated (V) and unvegetated (NV) habitats in the studied estuaries. Sites: BEN, Benevente estuary, VIB, Vitória Bay estuary, PAE, Piraque-Açu-Mirim estuary.

The macrofaunal assemblage composition was significantly different in several spatial scales within the mesohaline sector of the three estuaries (PERMANOVA; Table 4). These significant differences occurred in the interaction among habitat and all the spatial scales analyzed (estuary, site and plot) and the spatial scales within estuaries (site and plot). Faunal distribution patterns in nMDS ordination evidenced differences between unvegetated and vegetated habitats, with more heterogeneous assemblages in the former if compared to tightly grouped vegetated samples (Fig. 6). This pattern of higher spatial variability was also observed among the three estuaries, where macrofaunal assemblages at unvegetated habitats had lower similarity if compared to vegetated habitats.

**Table 4:** PERMANOVA results calculated from the Bray–Curtis dissimilarity matrix for the macrofauna communities at the different scales investigated of the three estuaries BEN, PAE and VIB.

Source	*df*	MS	Pseudo-*F*	P (perm)
H	1	34,861	1.895	0.23
E	2	24,587	5.153	0.06
S(E)	3	4,771.7	3.025	0.0001^*^
H × E	2	18,394	4.366	0.02^*^
P(S(E))	12	1,577.5	2.414	0.0001^*^
H × S(E)	3	4,213.5	2.593	0.0016^*^
H × P(S(E))	12	1,625	2.486	0.0001^*^
Residual	72	653.61		

**Notes.**

H habitat E estuary S site P plot

* Significant values.

Dissimilarities were high (>60%) between habitats inside each estuary and among estuaries in the unvegetated habitat (SIMPER). Kalliapseudidae, Oligochaeta, Capitellidae and Ampharetidae were the taxa that most contributed to the observed differences among habitats in the mesohaline sector of BEN (SIMPER; Table A1). At VIB and PAE, Oligochaeta, Spionidae, Capitellidae, Nereididae and Pilargidae were the taxa that most contributed to the observed differences among habitats in the mesohaline sector (SIMPER; Table A1). The dissimilarity between unvegetated habitats among estuaries within the mesohaline sectors occurred mainly due to differences in abundance of Kalliapseudidae (BEN), Spionidae (VIB) and Oligochaeta (PAE; SIMPER; Table A2).

### Relationships between sediment properties and macrofaunal assemblages

**Figure 7:**
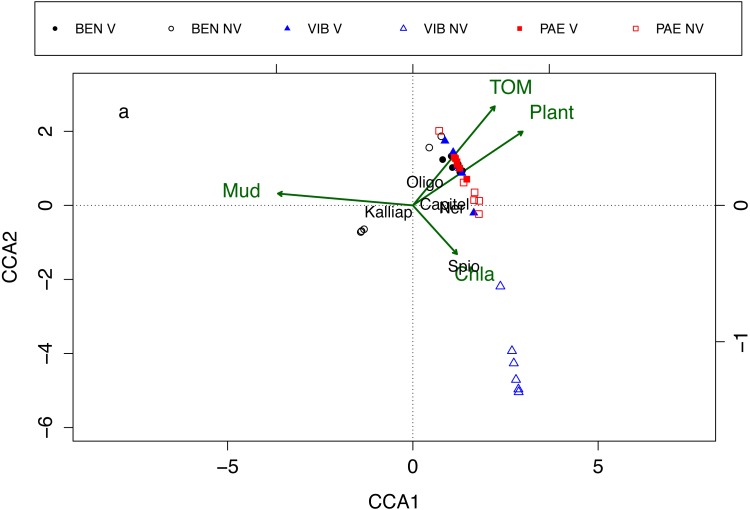
Canonical correspondence analysis of macrofaunal assemblages and environmental variables. Canonical correspondence analysis (CCA) for most abundant taxa (in black) and environmental properties (in green). Macrofaunal taxa indicated: Kalliap, Kalliapseudidae; Capitel, Capitellidae; Ner, Nereididae; Spio, Spionidae; Oligo, Oligochaeta. Environmental variables: TOM, total organic matter; Mud, mud content; Plant, plant biomass; Chla, Chlorophyll-a. Sites: BEN, Benevente estuary; VIB, Vitória Bay estuary; PAE, Piraque-Açu-Mirim estuary. V, vegetated habitat; NV, unvegetated habitat.

Macrofaunal assemblages were related to sediment mud content, TOM, plant biomass and Chl*-a*, with the first and second canonical axes explaining 26% and 17.2% of the variation in the data, respectively (CCA; Fig. 7). These relationships also explained the differences in assemblage composition between vegetated and unvegetated habitats. The CCA evidenced differences between habitats and estuaries and three groups of samples were formed in the CCA. The first group was corresponding to unvegetated habitat in VIB, the second group to unvegetated habitats in BEN, and the third group was formed by samples from both habitats in PAE, vegetated habitat in VIB and in BEN. Vegetated habitats of the three estuaries were related to higher TOM content, higher plant biomass and to higher densities of Oligochaeta and Capitellidae. Nereididae was also a family with high densities at vegetated habitats in PAE. Unvegetated habitats were more heterogeneous between estuaries, with VIB exhibiting higher Chl*-a* and dominated by Spionidae, whereas at PAE Capitellidae was dominant. At BEN, Kalliapseudidae was abundant at unvegetated sediments with high mud content and relative low plant biomass and TOM content.

## Discussion

Macrofaunal assemblage composition, abundance and secondary production exhibited different patterns of spatial variability within the three estuaries on the Eastern Brazil Marine Ecoregion. We observed marked differences in macrofaunal densities between the estuaries, but with inconsistent patterns between vegetated and unvegetated habitats. At the BEN estuary, spatial differences included a high dominance of Kalliapseudidae in unvegetated habitats in a similar pattern with subtropical estuaries (Lana & Guiss, 1991; Leite, Turra & Souza, 2003; Pagliosa & Barbosa, 2006; Pennafirme & Soares-Gomes, 2009). However, tanaidaceans were not sampled at the PAE and were very rare at VIB estuaries, suggesting that they may be occasional opportunists on tidal flats (Nucci, Turra & Morgado, 2001; Leite, Turra & Souza, 2003). In contrast to our hypothesis, vegetated and unvegetated habitats at PAE and VIB estuaries had similar macrofaunal densities, supporting that abundance is not strictly related to the presence or absence of vegetation (Schrijvers & Vincx, 1995; Sheridan, 1997; Yu et al., 1997; Alfaro, 2006). As observed elsewhere, macrofaunal densities can be highly variable between estuaries and among estuarine habitats and the macrofaunal abundances from Eastern Brazil estuaries are in the range of values of other tropical and temperate ecosystems (Table A3).

Macrofaunal assemblage composition had higher similarity within mangrove forests if compared to tidal flat assemblages. Mangrove associated fauna were composed mainly by Oligochaeta and Capitellidae despite the differences in urbanization among estuaries. These taxa are typically dominant in sediments with high organic content and detritus, and are widely present at other tropical and subtropical mangroves (Schrijvers & Vincx, 1995; Sheridan, 1997; Netto & Lana, 1999; Dittmann, 2001; Netto & Galluci, 2003; Demopoulos & Smith, 2010). Mangrove derived detritus and sedimentation patterns in nearby sediments can also have indirect effects in the composition and abundance of macrofauna (Netto & Lana, 1999; Netto & Galluci, 2003; Sweetman et al., 2010; Bernardino et al., 2018).

Macrofaunal estuarine assemblages may change in response to variable levels of disturbance (Lindegarth & Hoskin, 2001). The three sampled estuaries have wide differences in ecosystem quality, suggesting that habitat dissimilarity between estuaries were mostly related to local impacts, including pollution. For example, Kalliapseudidae was a dominant group in tidal flats of BEN estuary suggesting higher estuarine ecosystem quality (Pagliosa & Barbosa, 2006). However, Spionidae and Capitellidae were dominant both in VIB and PAE estuaries. VIB is a heavily polluted region whereas the PAE estuary is located in a conservation area, but still with detectable organic pollutants (Grilo et al., 2013). As a result, the macrofaunal assemblage composition of the three estuaries include a broad range of tolerant (pollution), rare and opportunist taxa in response to multiple ecosystem changes, both natural and human. Given variable levels of local impacts, we could not identify consistent patterns of benthic macrofaunal assemblages from intertidal vegetated and unvegetated habitats as recently observed for subtidal habitats in Eastern Brazil (Barros et al., 2012; Mariano & Barros, 2014).

The density and composition of macrofauna varied at local spatial scales within estuaries (among plots and also in the interaction between habitat and plot), indicating a patchy distribution (Underwood & Chapman, 1996; Underwood, Chapman & Connell, 2000; Chapman & Tolhurst, 2004; Morais, Camargo & Lana, 2016). Mean grain size, mud content, TOM and plant biomass also varied at the same spatial scales, and likely influenced macrofaunal assemblages.

In general, estuarine macrofaunal biomass in the Eastern Brazil Marine Ecoregion was comparable to other temperate estuaries (Table A3). Macrofaunal biomass and secondary production were higher in vegetated habitats in the mesohaline sector at PAE and VIB, suggesting that mangrove forests are an important source of organic material to the local benthic assemblages (Edgar, 1990b; Sprung, 1994; Heck et al., 1995; Dolbeth et al., 2003; Bernardino et al., 2018). However, habitat structure may also increase benthic biomass and secondary production by creating microhabitats and offering protection from predators (Edgar, 1990b; Kon, Kurokura & Tongnunui, 2010). These differences may be important at regional scales, creating significant changes in benthic secondary production among estuaries. In our study, higher biomass and production at unvegetated habitats occurred due to the high densities of Kalliapseudidae, which have continuous reproduction and fast growth (Fonseca & D’Incao, 2003; Leite, Turra & Souza, 2003; Pennafirme & Soares-Gomes, 2009). So, it seems that regional changes in the composition of benthic assemblages may also temporally lead to significant changes in benthic production, and long-term studies could help identify seasonal and inter-annual patterns (Dolbeth et al., 2003).

Macrofaunal biomass and production were driven by variable taxonomic groups and size classes. Unvegetated habitats at BEN estuary had higher biomass and production given high Kalliapseudidae densities. These tanaidaceans are deposit and suspension feeders and offer direct trophic links to fishes, birds and other crustaceans (Lana & Guiss, 1991; Pagliosa & Barbosa, 2006; Pennafirme & Soares-Gomes, 2009), suggesting its importance to estuarine food webs at BEN. Other mollusks and crustaceans markedly contributed to total biomass and production despite relative low densities in vegetated and unvegetated habitats. Mytilidae contributed to mangrove benthic biomass at VIB and PAE estuaries, and are important human food resources (Nishida & Leonel, 1995; Nalesso et al., 2005). Brachyurans were also important to biomass and production of mangrove sediments suggesting their importance as a food source and to overall ecosystem health (Koch & Wolff, 2002; Cannicci et al., 2008).

The benthic biomass turnover rate (P/B ratio) was variable between habitats and estuaries. At PAE and VIB estuaries, the P/B ratio was higher or slightly higher in unvegetated habitats suggesting higher turnover rates of benthic production at tidal flats (Edgar et al., 1994; Sprung, 1993; Sprung, 1994). The lower P/B ratio in vegetated habitats occurred due to the dominance in biomass and production of bivalves and crustaceans (crabs) that were larger individuals with slow growth rates and longer life spans (Edgar & Barrett, 2002). At BEN estuary, P/B ratio was relatively similar between tidal flats and mangroves. As higher P/B ratios suggest higher population resilience to environmental perturbations (Tumbiolo & Downing, 1994), highly productive estuarine habitats including tidal flats at BEN estuary may indicate target areas for estuarine conservation in Eastern Brazil. Although our results are limited by lack of temporal analysis, the empirical models applied to a single sampling campaign may show relative differences between estuarine regions under similar climatic conditions (Edgar, 1990a; Tumbiolo & Downing, 1994; Brey, 2001; Dolbeth et al., 2012). For example, although P/B ratio did not show consistent spatial variability among estuaries, the mangrove forests supported a similar benthic production within the three estuaries suggesting a great resilience of invertebrate assemblages to pollution and other impacts.

The Eastern Brazil Marine Ecoregion is experiencing loss of mangrove forests and multiple other impacts to estuaries (Barros et al., 2012; Bernardino et al., 2015; Gomes et al., 2017; Bernardino et al., 2018; Servino, Gomes & Bernardino, 2018). As in other estuarine ecosystems, macrofaunal assemblages are highly variable with respect to taxa composition and abundance. However, our data suggest that secondary production, which is a measure of ecosystem function yet poorly evaluated in most estuaries, may provide an important information of ecosystem change that could be used to track ecosystem health and indicate management actions towards areas with higher ecosystem quality (Dolbeth et al., 2011; Dolbeth et al., 2012; Vilar, Joyeux & Spach, 2017). The implementation of long-term monitoring series that includes macrofaunal secondary production would markedly increase our understanding of estuarine ecosystem functioning in Eastern Brazil.

## Conclusions

In summary, we found that macrofaunal assemblages varied at multiple spatial scales, between vegetated and unvegetated habitats and among estuaries. Macrofaunal density varied at the scale of individual samples, whereas biomass and secondary production differed between the interaction of habitats and estuary suggesting that estuarine benthic ecosystem functioning varies markedly at regional scales. Mangrove and tidal flat habitats had distinct patterns of production to biomass ratio, with larger individuals with longer time spans at vegetated habitats which may promote higher resilience to environmental perturbations in urban estuaries in Eastern Brazil. Benthic secondary production may offer an alternative metric to evaluate estuarine ecosystem health among estuaries in Eastern Brazil, and should be incorporated in long-term assessments to support management of local impacts and future climate change effects.

##  Supplemental Information

10.7717/peerj.4441/supp-1 Supplemental Information 1 Raw data including environmental and biological datasets

10.7717/peerj.4441/supp-2 Supplemental Information 2 Appendix figures and tables

## Additional Information and Declarations

### Field Study Permissions

The following information was supplied relating to field study approvals (i.e., approving body and any reference numbers): Collection of fauna was approved by the Brazilian Ministry of Environment (ICMBIO).

### Data Availability

The following information was supplied regarding data availability: The raw data are provided as a Supplemental File.
