# Supplementary material for: Benthic macrofaunal structure and secondary production in tropical estuaries on the Eastern Marine Ecoregion of Brazil
Source: PeerJ. 2018 Feb 28;6:e4441. doi: 10.7717/peerj.4441 (PMC5834938; doi:10.7717/peerj.4441)
Supplement: Supplemental Information 2 [file peerj-06-4441-s002.docx]

**Appendix**


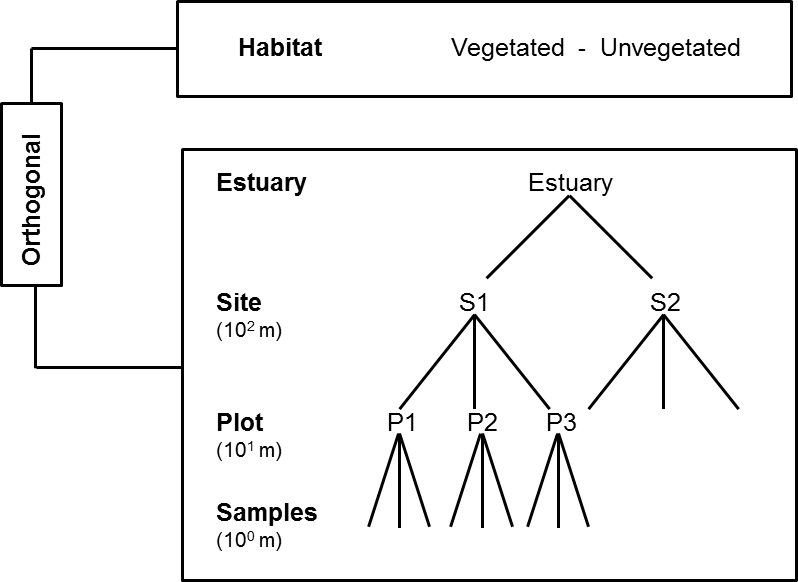


Fig. A1. Sampling design diagram with spatial and habitat scales that correspond to the factors of the ANOVA: Habitats (V, NV) orthogonal to spatial nested scales of Estuaries (BEN, VIB, PAE), Sites (S1, S2) and Plots (P1, P2, P3), with three replicates each.

Table A1. Similarity percentages (SIMPER) analysis with contribution (%) for each taxa that most contributed to dissimilarities among habitats at the mesohaline sector in the estuaries at the cutoff level of 80%.

| **BEN V - BEN NV** |  |  |  |
| --- | --- | --- | --- |
| **Species** | **Av.Diss** | **Contrib%** | **Cum.%** |
| **Kalliapseudidae** | 39.24 | 51.95 | 51.95 |
| **Oligochaeta** | 12.36 | 16.36 | 68.31 |
| **Capitellidae** | 3.8 | 5.03 | 73.34 |
| **Ampharetidae** | 3.79 | 5.02 | 78.36 |
| **Polychaeta sp1** | 3.4 | 4.5 | 82.86 |
|  |  |  |  |
| **PAE V - PAE NV** |  |  |  |
| **Species** | **Av.Diss** | **Contrib%** | **Cum.%** |
| **Oligochaeta** | 20.02 | 27.98 | 27.98 |
| **Capitellidae** | 13.2 | 18.44 | 46.42 |
| **Nereididae** | 7.22 | 10.09 | 56.51 |
| **Pilargidae** | 4.21 | 5.89 | 62.4 |
| **Ampharetidae** | 4.07 | 5.69 | 68.09 |
| **Nemertea** | 3.89 | 5.44 | 73.52 |
| **Bivalvia not ID** | 2.64 | 3.69 | 77.21 |
| **Spionidae** | 2.63 | 3.67 | 80.88 |
|  |  |  |  |
| **VIB V - VIB NV** |  |  |  |
| **Species** | **Av.Diss** | **Contrib%** | **Cum.%** |
| **Oligochaeta** | 21.25 | 32.28 | 32.28 |
| **Spionidae** | 10.31 | 15.66 | 47.94 |
| **Capitellidae** | 7.4 | 11.24 | 59.18 |
| **Nereididae** | 5.72 | 8.69 | 67.87 |
| **Pilargidae** | 2.64 | 4.01 | 71.88 |
| **Polychaeta sp2** | 2.2 | 3.34 | 75.21 |
| **Bivalvia not ID** | 2.09 | 3.18 | 78.39 |
| **Sipuncula** | 1.95 | 2.96 | 81.35 |

Table A2. Similarity percentages (SIMPER) analysis with contribution (%) for each taxa that most contributed to dissimilarities in NV habitats among estuaries at the cutoff level of 80%.

| **BEN NV – PAE NV** | | | |  |  | **BEN NV – VIB NV** | | | |
| --- | --- | --- | --- | --- | --- | --- | --- | --- | --- |
| **Species** | **Av.Diss** | **Contrib%** | **Cum.%** |  |  | **Species** | **Av.Diss** | **Contrib%** | **Cum.%** |
| **Kalliapseudidae** | 49.96 | 56.29 | 56.29 |  |  | **Kalliapseudidae** | 41.34 | 48.25 | 48.25 |
| **Oligochaeta** | 15.05 | 16.95 | 73.24 |  |  | **Spionidae** | 13.7 | 15.99 | 64.24 |
| **Capitellidae** | 5.24 | 5.91 | 79.15 |  |  | **Oligochaeta** | 12.6 | 14.71 | 78.95 |
| **Nereididae** | 3.6 | 4.05 | 83.2 |  |  | **Capitellidae** | 3.48 | 4.06 | 83.01 |
|  |  |  |  |  |  |  |  |  |  |
| **VIB NV - PAE NV** | | | |  |  |  | | | |
| **Species** | **Av.Diss** | **Contrib%** | **Cum.%** |  |  |  |  |  |  |
| **Spionidae** | 30.31 | 38.92 | 38.92 |  |  |  |  |  |  |
| **Capitellidae** | 11.2 | 14.38 | 53.29 |  |  |  |  |  |  |
| **Nereididae** | 7.96 | 10.22 | 63.51 |  |  |  |  |  |  |
| **Oligochaeta** | 5.61 | 7.2 | 70.71 |  |  |  |  |  |  |
| **Bivalvia not ID** | 4.65 | 5.97 | 76.69 |  |  |  |  |  |  |
| **Pilargidae** | 4.43 | 5.69 | 82.37 |  |  |  |  |  |  |

Table A3. Macrofaunal densities (ind.m-2) reported in mangroves and tidal flats in different tropical (*), subtropical (**) and temperate (***) estuaries. Min.= minimum value; max.= maximum value.

| **Location** | **Macrofaunal density**  **(min. – max.)**  **(ind.m^−2^)** | **Macrofaunal biomass (min. – max.)**  **(g AFDW m^-2^)** | **Macrofaunal secondary production (min. – max.) (mg m^-2^ day^-1^)** | **Habitat** | **Mesh size (mm)** | **Reference** | |
| --- | --- | --- | --- | --- | --- | --- | --- |
| Rio Grande Marine  Ecoregion of Brazil | Up to 7,250 |  |  | Mangrove** | 0.5 | Netto & Galluci 2003 | |
| Kenya | 265 to 4,125 (mean of 1,933) |  |  | Mangrove* | 1 | Schrijvers et al.  1995 | |
| Florida | 22,591 to 52,914 |  |  | Mangrove** | 0.5 | Sheridan 1997 | |
| New Zealand | Approx. 8,000 |  |  | Mangrove*** | 0.5 | Alfaro 2006 | |
| Eastern Brazil Marine  Ecoregion | < 1,000 to 6,000 |  |  | Tidal flats* | 0.5 | Mariano & Barros, 2014 | |
| Southeastern Brazil Marine  Ecoregion | 500 to 3,000 |  |  | Tidal flats** | 0.5 | Morais et al.  2016 | |
| England (UK) |  | average 8.65 |  | Tidal flats*** | 0.86 | Fujii 2007 | |
| NW Europe (Netherlands and Belgium) | 0 to 225,568 | 0 to 466.5 |  | Tidal flats*** | 1 | Ysebaert et al. 2003 | |
| New Zealand |  | approximately 1 | approximately 10 | Intertidal estuary *** | 0.5 to 8.0 | Cowles et al. 2009 | |
| Eastern Brazil Marine | 3,013 (847 - 10,904) | 2.1805 | 38.3 | Mangrove BEN* | 1 mm | Present study | |
| Ecoregion | 11,802 (2,655 - 27,740) | 2.8302 | 58.4 | Mangrove VIB* |  |  | |
|  | 4,391 (1,695 - 8,983) | 4.466 | 65 | Mangrove PAE* |  |  | |
| (Mean values) | 33,023 (1,808 – 60,056) | 7.6774 | 137.8 | Tidal flats BEN* |  |  | |
|  | 3,349 (452 – 7,458) | 0.53 | 13.5 | Tidal flats VIB* |  |  | |
|  | 1,033 (0 – 6,554) | 0.1007 | 3.5 | Tidal flats PAE* |  |  |  |

**References**

Alfaro AC. 2006. Benthic macro-invertebrate community composition within a mangrove/seagrass estuary in northern New Zealand. Estuarine Coastal and Shelf Science 66:97–110

Cowles A, Hewitt JE, Taylor RB. 2009. Density, biomass and productivity of small mobile invertebrates in a wide range of coastal habitats. Marine Ecology Progress Series 384:175–185

Fujii T. 2007. Spatial patterns of benthic macrofauna in relation to environmental variables in an intertidal habitat in the Humber estuary, UK: Developing a tool for estuarine shoreline management. Estuarine Coastal and Shelf Science 75:101-119

Mariano DLS, Barros F. 2014. Intertidal benthic macrofaunal assemblages: changes in structure along entire tropical estuarine salinity gradients. Journal of the Marine Biological Association of the United Kingdom 95:5-15

Morais GC, Camargo MG, Lana P. 2016. Intertidal assemblage variation across a subtropical estuarine gradient: How good conceptual and empirical models are? Estuarine Coastal and Shelf Science 170:91-101

Netto SA, Galluci F. 2003. Meiofauna and macrofauna communities in a mangrove from the Island of Santa Catarina, South Brazil. Hydrobiologia 505:159–170

Schrijvers J, Gansbeke D Van Vincx M. 1995. Macrobenthic infauna of mangroves and surrounding beaches at Gazi Bay, Kenya. Hydrobiologia 306:53-66

Sheridan P. 1997. Benthos of adjacent mangrove, seagrass and non-vegetated habitats in Rookery Bay, Florida, U.S.A. Estuarine Coastal and Shelf Science 44:455–469

Ysebaert T, Herman PMJ, Meire P, Craeymeersch J, Verbeek H, Heip CHR. 2003. Large-scale spatial patterns in estuaries: estuarine macrobenthic communities in the Schelde estuary, NW Europe. Estuarine Coastal and Shelf Science 57:335-355
